# Rationale for COVID-19 Treatment by Nebulized Interferon-β-1b–Literature Review and Personal Preliminary Experience

**DOI:** 10.3389/fphar.2020.592543

**Published:** 2020-11-30

**Authors:** Aurélien Mary, Lucie Hénaut, Pierre Yves Macq, Louise Badoux, Arnaud Cappe, Thierry Porée, Myriam Eckes, Hervé Dupont, Michel Brazier

**Affiliations:** ^1^ Clinical Critical Care Pharmacy Department, Amiens-Picardie University Hospital, Amiens, France; ^2^ UR UPJV 7517, MP3CV, CURS, University of Picardie Jules Verne, Amiens, France; ^3^ Surgical Critical Care Department, Amiens-Picardie University Hospital, Amiens, France; ^4^ ProtecSom-OptimHal, Valognes, France; ^5^ Department of Biochemistry, Amiens-Picardie University Hospital, Amiens, France

**Keywords:** COVID-19, SARS-CoV-2, nebulization, inflammation, interferon-β-1b

## Abstract

The inflammatory response to COVID-19 is specifically associated with an impaired type I interferon (IFN) response and complete blockade of IFN-β secretion. Clinically, nebulization of IFN-α-2b has been historically used in China to treat viral pneumonia associated with SARS-CoV. Very recent data show that the use of inhaled type I IFN is associated with decreased mortality in Chinese COVID-19 patients. However, IFN nebulization is currently not standard in Europe and the United States. Therefore, our group has set up a project aimed to evaluate the possibility to nebulize IFN-β-1b (a drug currently used in Europe to treat multiple sclerosis via subcutaneous injections) and to assess the safety of this new mode of administration in SARS-CoV-2 infected patients. We present here literature data that allowed us to build our hypothesis and to develop collaboration between clinical pharmacists, intensivists and nebulization engineers in order to gain first pre-clinical and clinical experience of IFN-β-1b nebulization. After validation of the nebulization method and verification of droplet size compatible with nebulization, the method has been applied to four intensive care patients treated at our university hospital, for whom none of the COVID-19 therapies initially used in France led to significant clinical improvement. All patients exhibited negative viral carriage and experienced clinical improvement 7–16 days after having initiated nebulized IFN-β-1b inhalation therapy. No side effects were observed. All patients were alive within a 90-days follow-up. Although it is not possible to draw firm conclusions on treatment efficacy based on this case report, our study shows that pulmonary IFN-β-1b administration is feasible, with a good safety profile. This procedure, which presents the advantage of directly targeting the lungs and reducing the risks of systemic side effects, may represent a promising therapeutic strategy for the care of patients with severe COVID-19. However, our preliminary observation requires confirmation by randomized controlled trials.

## Introduction

The emergence of SARS-CoV-2 and the resulting COVID-19 pandemic has caused the most serious health crisis of this century. SARS-CoV-2 is responsible for respiratory disease which frequently leads to severe forms of pneumonia and acute respiratory distress syndromes (ARDS) (Zhu et al., 2020). In addition, many other vital organs may be damaged as well. Overall case-fatality is estimated to be around 1%, but can reach 25–60% in patients requiring intensive care (Armstrong et al., 2020; Quah et al., 2020; Wu and McGoogan, 2020). The mortality risk depends on many different factors, including advanced age, male gender and presence of comorbidities such as chronic obstructive pulmonary disease (COPD), obesity, diabetes, cardiovascular disease, kidney disease and cancer (Docherty et al., 2020; Parohan et al., 2020), and last but not least type of patient management (Gupta et al., 2020).

The pathological process can be explained by a two-phase course of SARS-CoV-2 infection. The first phase corresponds to viral proliferation, associated with contamination of the bronchial tree by an interaction between the spike proteins of the virus and ACE2 receptors (Hoffmann et al., 2020). Beyond the viral infection per se, accumulating evidence suggests that a subgroup of patients with severe COVID-19 develop a severe inflammatory syndrome characterized by a dramatic rise of circulating pro-inflammatory cytokines IFN-γ, TNF-α, IL-6, IL-1 (Yang A.-P. et al., 2020; Yang L. et al., 2020) that attack cells in the pulmonary alveoli (Bradley et al., 2020; Carsana et al., 2020). The inflammatory reaction associated with SARS-CoV-2 infection contributes to the severity of the disease, and is associated with thrombotic and respiratory manifestations that require, in the most severely affected patients, intensive oxygen therapy, or even intubation leading to prolonged stays in intensive care units (Yuki et al., 2020). The viral invasion and inflammation may also be responsible for a multisystem disease, including cardiac, renal and neurological damage (Zhang B. et al., 2020; Chen T. et al., 2020; Helms et al., 2020).

To date, the management of patients showing the most severe forms of the disease remains essentially symptomatic and mainly consists in supply of oxygen (Phua et al., 2020), preventive anticoagulation adapted to disease severity (Atallah et al., 2020), and the use of low-dose corticosteroid therapy (RECOVERY Collaborative Group et al., 2020a). Despite intensive research and debate about the most efficient therapeutic approach leading to decreased viral shedding and improved patient outcomes, no antiviral treatment has as yet been convincingly shown to reduce COVID-19 mortality, be it hydroxychloroquine (hCQ) (RECOVERY Collaborative Group et al., 2020b; Fiolet et al., 2020), remdesivir (Beigel et al., 2020) or lopinavir/ritonavir (Cao et al., 2020; RECOVERY Collaborative Group, 2020; WHO, 2020).

**FIGURE 1:**
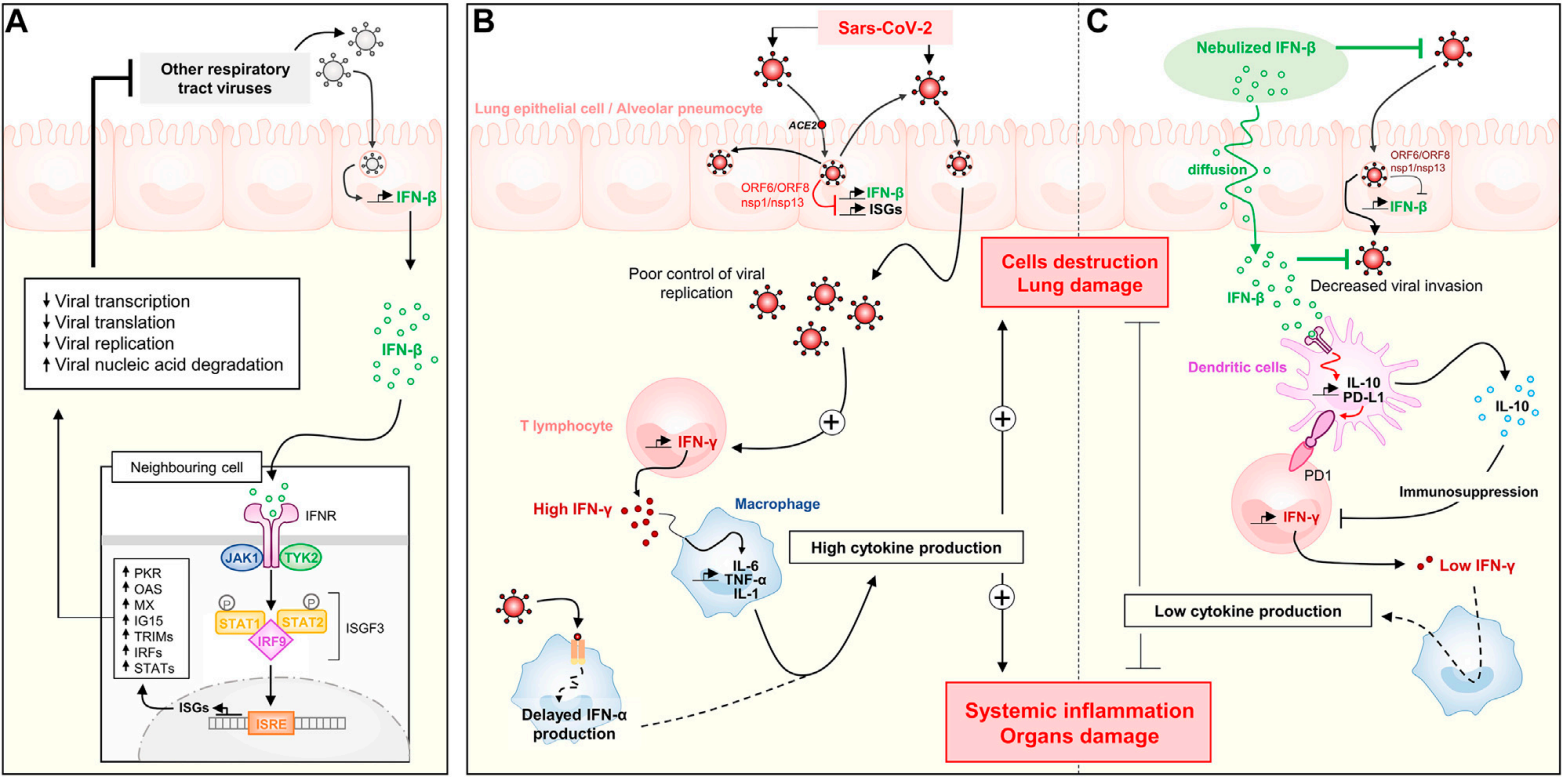
Type I interferon response and the reduction of viral shedding: the impact of SARS-CoV-2 and key role of inhaled IFN-β. **(A)** Usual response to a respiratory virus such as influenza. **(B)** Immune response described in the severe COVID-19, with impaired response to type I IFN and excessive pro-inflammatory cytokine production. **(C)** Hypothesis on the therapeutic value of IFN-β nebulization on viral control and inflammation of COVID-19. IFN, Interferon; IL, Interleukine; TNF, Tumor Necrosis Factor; ISG, Interferon-stimulated genes; ISRE, Interferon-stimulated response element.

In this context, our group highlighted early on promising signals from China as regards the potential therapeutic interest of pulmonary type I interferon (IFN) administration (Mary et al., 2020). Type I IFNs are cytokines which represent a major innate anti-viral defense. Type I IFN includes IFN-α, mainly produced by macrophages, and IFN-β produced by bronchial epithelial cells in response to viral infection. They display the ability to bind the surface of infected and neighboring cells and promote the induction of more than 1,000 different IFN-inducible genes (ISGs) that ultimately prevent virus protein trafficking, virus RNA synthesis or virion assembly and release (Hesse et al., 2009; Rauch et al., 2013; Schreiber and Piehler, 2015) (Figure 1A).

Although the use of IFN-α-2b nebulization is widespread in China for the control of respiratory viral diseases (Mary et al., 2020) currently such nebulization is not standard in Europe and United States. Moreover, IFN-α-2b is no longer marketed in many European countries since the end of 2019. Therefore, our group set up a project aimed to evaluate the possibility of administering nebulized IFN-β-1b by inhalation and to assess the safety of this new route of administration in SARS-CoV-2 infected patients. Note that IFN-β-1b is currently used in Europe to treat multiple sclerosis via sub-cutaneous injection. The present manuscript reports how a collaborative effort involving a clinical pharmacy department and an intensive care unit enabled the treatment of four patients suffering from severe COVID-19 with inhaled IFN-β-1b, as well as investigations carried out in collaboration with an industry company to better characterize this off-label route of administration.

The first section of the manuscript is devoted to literature reports that drove us to develop the hypothesis that type I IFN inhalation (and in particular INF-β) may reduce the duration of viral carriage and improve major symptoms in COVID-19 patients. The second part of the manuscript presents *in vitro* data on the feasibility of IFN-β-1b nebulization. In this section, we discuss the choice of the nebulization solvent and excipients, and present data on the stability of the product once reconstituted. We then report the aerodynamic size of particles obtained when the solution of IFN-β-1b was nebulized in a cascade impactor mimicking lung nebulization. The last part of the manuscript focuses on the clinical course of four intensive care patients, who received inhalation of IFN-β-1b as developed by our group, combined or not with lopinavir/ritonavir. This treatment was given on a compassionate basis after failure of the initial COVID-19 therapy (including antibiotic treatment with cefotaxime/azithromycin either alone or combined with hCQ). The final discussion focuses on the achievement of therapeutic targets with the inhalation system, as well as future prospects to better determine the potential therapeutic role of inhaled IFN in the treatment of COVID-19.

## Type I Interferon Response and COVID-19: A Review of the Literature

### COVID-19 Patients Show a Delayed Type I Interferon Response

Since our first report (Mary et al., 2020) a variety of data have been published, presenting additional arguments supporting the usefulness of the type I IFN pathway in treating COVID-19 patients. In particular, an impaired type I IFNs response in lung tissue has been identified in COVID-19 and analyzed in detail (Park and Iwasaki, 2020).

In fact, Blanco-Melo et al. have shown that SARS-CoV-2 induced only a very weak type I and III IFN response, juxtaposed to a dramatic inflammation characterized by excessive serum IL-6 and TNF-α levels (Blanco-Melo et al., 2020) (Figure 1B). Such a weak type I IFN response has also been reported by others (Trouillet-Assant et al., 2020a; Hadjadj et al., 2020). It is associated both with longer carriage time and severity of inflammation. This suggests compensation for type I IFN deficit by other immunological routes probably responsible for the frequent observation of a cytokine storm. It should be noted that in these three studies, serum concentrations of IFN-β were undetectable in COVID-19 patients (Trouillet-Assant et al., 2020a; Blanco-Melo et al., 2020; Hadjadj et al., 2020), suggesting absent IFN secretion by the pulmonary epithelia.

This has been confirmed in Calu3 cells, primary human airway epithelial cells (pHAE), alveolar epithelial type 2 cells (AT2s), A549 lung alveolar cells and in a reconstituted human bronchial epithelium model (MucilAir™ model), where SARS-CoV-2 infection failed to induce an appropriate type I and III IFN response (Blanco-Melo et al., 2020; Huang et al., 2020; Robinot et al., 2020; Shuai et al., 2020; Vanderheiden et al., 2020). According to recent reports, COVID-19 also causes an impaired type I IFN response in the periphery. Indeed, Arunachalam et al. showed reduced production of IFN-α in response to TLR stimulation in the plasmacytoid dendritic cells of infected individuals compared with those of healthy controls (Arunachalam et al., 2020), which suggests that plasmacytoid dendritic cells, the primary producers of type I IFNs, are impaired in COVID-19 infection.

SARS-CoV-2 has developed several mechanisms to hijack the innate immune system via both its structural and non-structural proteins. Among them, viral ORF3b, ORF6, ORF7a, ORF7b, ORF8, ORF9b, nsp1, nsp3, nsp5, nsp6, nsp12, nsp13, nsp14, nsp15, nucleocapsid (N) and membrane (M) proteins were reported to be potential inhibitors of type I interferon production and/or type I IFN signaling pathway (Jiang H.-W. et al., 2020; Konno et al., 2020; Lei et al., 2020, 2; Li et al., 2020; Mu et al., 2020; Wu et al., 2020; Xia et al., 2020; Yuen et al., 2020). Viral ORF6 showed the most potent inhibition on IFN-β promoter and also inhibits the interferon-stimulated response element after Sendai virus infection (Lei et al., 2020, 2; Li et al., 2020; Xia et al., 2020; Yuen et al., 2020). Recently, Thoms et al. demonstrated the role of SARS-CoV-2 nsp1 protein in evading the immune system, including its ability to totally abrogate the ribosomal translation induced by IFN-β promoters (Thoms et al., 2020). The impaired IFN-β response and the specific type of inflammation induced by Sars-CoV-2 should be analyzed taking into account the capacity of IFN-β to exert immunosuppressive actions, as observed in multiple sclerosis (Reder and Feng, 2014). Specifically, type I IFNs are able to suppress inflammatory cytokine release, by decreasing IFN-γ production and by promoting IL-10 production (McNab et al., 2014). The hypothesis of compensating the IFN-β deficiency of pulmonary origin by excessive lymphocyte IFN-γ production could help to solve the apparent antinomy with the inflammatory transcriptomic profile of monocytes and lymphocytes (with a marked TNF-α and IL-1β signature and an unconstant ISG signature) (Gardinassi et al., 2020; Lee et al., 2020; Maucourant et al., 2020; Wen et al., 2020; Wilk et al., 2020).

The dramatic pulmonary SARS-CoV-2 invasion observed in deceased COVID-19 patients (Bradley et al., 2020; Hanley et al., 2020), reinforces the hypothesis that the disease severity is associated with an immune system failure to clear the virus. In line with this hypothesis it has been shown that the response to type I IFNs is impaired in subgroups of patients at high risk of mortality from COVID-19. This is the case of elderly people (Abb et al., 1984), especially men with an “X chromosome monosomy (XCM)” linked to Y chromosome loss (Perez-Jurado et al., 2020). Interestingly Zhao et al. observed an impressive association between administration of inhaled IFN-α-2b and survival in people over 65 years of age (0.29 [95% CI 0.17–0.51], *p* < 0.001), suggesting that the elderly might represent a subgroup of patient particularly responsive to the treatment (Mei et al., 2020). Similarly, obese patients at risk for severe COVID-19 are known to have delayed responses to type I IFN (Teran-Cabanillas et al., 2013). In contrast, the mild forms of COVID-19 seen in children are likely to be related to their ability to produce type I IFN more efficiently than elderly persons (Trouillet-Assant et al., 2020b). In accordance with these data, type I IFN signaling pathway has been shown to be significantly up-regulated in lungs of juvenile macaques infected with SARS-CoV2, as compared to old infected macaques (Rosa et al., 2020). In SARS-CoV-infected aged macaques, the severity of the disease is associated with an increase in the expression of genes associated with inflammation and reduced expression of IFN-β as compared to young adult animals, who display a mild form of the disease (Smits et al., 2010). Treatment of old macaques with type I IFN reduced pro-inflammatory gene expression and disease severity.

On the other hand, genetic mutations may explain specific susceptibility to COVID-19 dependent on IFN pathways. This is true for mutations in genes encoding interferon-induced transmembrane proteins type 3 (IFITM3) (Zhang Y. et al., 2020), i.e., vesicular proteins promoting viral trafficking to lysosomes (Spence et al., 2019). It has also been suggested that the black African population is more sensitive to SARS-CoV-2 due to a polymorphism in the gene encoding IFIH1 (InterFeron-Induced helicase 1), a host protein that senses the presence of viral RNA and subsequently promotes IFN production (Maiti, 2020). Interestingly, van der Made et al. found a unique loss-of-function variants in X-chromosomal TLR7 (with an impaired transcriptional host type I IFN response downstream) responsible of severe COVID-19 in four young men (van der Made et al., 2020). More recently, Zhang et al. identified inborn errors of TLR3-and IRF7-dependent type I IFN immunity in 3.9% patients with life-threatening COVID-19 pneumonia (Zhang Q. et al., 2020). *In vitro*, human fibroblasts with mutations affecting this pathway are vulnerable to SARS-CoV-2, suggesting that inborn errors of TLR3-and IRF7-dependent type I immunity can underlie critical COVID-19. Other genetic studies are underway to assess potential associations between patient genotypes and COVID-19 severity. They could determine the place of others polymorphisms of IFN pathways in the severity of COVID-19 (COVID-19 Host Genetics Initiative, 2020).

Beyond genetic susceptibility, a study recently reported in *Science* showed that 13.7% of patients with life-threatening COVID-19 pneumonia have autoantibodies against IFN-ω and/or IFN-α able to neutralize the ability of the corresponding type I IFNs to block SARS-CoV-2 infection (Bastard et al., 2020, 19). Interestingly, only 1.9% of these critical patients had IFN-β autoantibodies of which only 10% displayed neutralizing properties *in vitro*. Collectively, these data suggest that an early treatment with IFN-α is unlikely to be beneficial in this patient group. On the opposite, treatment with IFN-β may have beneficial effects, since autoantibodies against IFN-β appear to be rare in patients with autoantibodies directed against type I IFNs.

Collectively, the available experimental and clinical data make us hypothesize that COVID-19 severity is at least partially caused by an insufficient innate IFN-β response of the pulmonary epithelium. This defect is subsequently overcompensated by an excessive leukocyte response promoting (among others) the secretion of type II IFN at the origin of the described interferonopathy of COVID-19 (Figure 1B). IFN-β in particular could thus represent a solid etiological therapeutic path, as also proposed by the Belardelli team (Aricò et al., 2020) (Figure 1C). We therefore focused on the therapeutic approach of treating COVID-19 by IFN, in particular with the *β* isoform, and the way to optimize a clinical answer.

### *In Vitro* Efficacy of Type I Interferon in a SARS-CoV-2 Infected Cellular Model

Recent *in vitro* studies have confirmed the potential inhibitory effect of type I Interferon on SARS-CoV-2. In Vero cells, the virus is more sensitive to pretreatment with IFN-β than with IFN-α, with a particularly low EC50 at 0.76 IU/ml (Mantlo et al., 2020). Pretreatment of a reconstituted human bronchial epithelium (MucilAir™ model) with IFN-β is also efficient against Sars-CoV2 in decreasing viral RNA levels by two logs (Robinot et al., 2020). In a curative model, the inhibitory potency of IFN-β decreases over time, from 0.4 to 4.9 IU/ml after 48 h to an EC50 of 3.5–6.0 IU/ml at 96 h (Clementi et al., 2020). At 96 h, the maximum effect (variation of 10 CT) is only obtained for high concentrations, greater than or equal to 50 IU/ml (Clementi et al., 2020). Ruxolitinib, an inhibitor of IFN-triggered janus kinase 2 (JAK)/signal transducer and activator of transcription (STAT) signaling, enhances SARS-CoV-2 proliferation in IFN competent Calu three cells (Felgenhauer et al., 2020). In a comparative study, IFN-β-1b showed a most potent anti SARS-CoV-2 effect than IFN-β-1a in VeroE6 cells, characterized by higher affinity (EC50 = 31.2 IU/ml vs. 70.8 IU/ml, respectively) and a higher selectivity index (selectivity index: 1,602.2 vs. 706.2, respectively) (Yuan S. et al., 2020). In the more physiological primary human airway epithelial (pHAE) model, pretreatment or post-treatment with IFN-β at 100 IU/ml reduced viral load by more than 90% (Vanderheiden et al., 2020).

### Impact of Type I Interferon Subcutaneous Injections on COVID-19 Patients

Based on these observations, a therapeutic approach with IFN was selected in the Solidarity and Discovery clinical trials, using subcutaneous IFN-β-1a administration every 3 days. To date, an IFN treatment arm remains in Solidarity, together with remdesivir. In Hong Kong, the team of Hung et al. conducted an open label, randomized phase 2a trial in 127 patients with mild to moderate forms of COVID-19 using the triple combination of subcutaneous IFN-β-1a, lopinavir/ritonavir and ribavirin. They found a significant reduction in viral carriage with a median from 12 to 7 days, complete symptom remission with a median from 8 to 4 days, as well as a reduction in the length of hospital stay (Hung et al., 2020). In Tehran, Davoudi et al. evaluated in 81 severe patients the impact of subcutaneous injection of IFN-β-1a (three times weekly for two consecutive weeks) to the treatment composed of hCQ + lopinavir/ritonavir or atazanavir/ritonavir. They observed a significant decrease in mortality at 28 days (19% vs. 43.6% *p* = 0.015), and an increase in discharge rate at 14 days (66.7% vs. 43.6%) (OR = 2.5; 95% CI: 1.05–6.37) (Davoudi-Monfared et al., 2020). More recently, the same group evaluated in 66 adults with severe COVID-19 the impact of subcutaneous injection of IFN-β-1b (every other day for two consecutive weeks) compared to the national protocol medications (lopinavir/ritonavir or atazanavir/ritonavir plus hydroxychloroquine for 7–10 days) (Rahmani et al., 2020). They observed that the time to clinical improvement in the IFN group was significantly shorter than in the control group ([9 (6–10) vs. 11 (9–15) days respectively, *p* = 0.002, HR = 2.30; 95% CI: 1.33–3.39]) and that the percentage of discharged patients at 14 days was significantly higher in the IFN group than in the control group (78.79% vs. 54.55%, respectively; OR = 3.09; 95% CI: 1.05–9.11, *p* = 0.03). ICU admission rate in the IFN group was significantly lower than the control group (42.4% vs. 66.7%, *p* = 0.04). In this trial, the treatment with IFN-β-1b did not impact all-cause 28-days mortality (6.06% in the IFN group vs. 18.18% in the control group, *p* = 0.12). In these trials, the efficacy of IFN was more marked when administered early during the disease course. In addition, investigators from Cuba also presented (pre-published) encouraging data on the use of IFN-α-2b by subcutaneous route in a prospective observational study, but this study contained several confounders which were not corrected by multivariable analysis (Pereda et al., 2020).

### Toward the Use of Type I Interferon Via Inhalation

Subcutaneous injection of IFN-β is suitable for obtaining prolonged immunomodulatory effects, but allows achievement of a maximum concentration of at best 5 IU/ml, with a half-life of about 5 h (Munafo et al., 1998), (Salmon et al., 1996). Note that upon intravenous injection of IFN-α, the bioavailability reached at the pulmonary level is only around 50% (Greig et al., 1988). *In vitro* data suggest that maximum concentrations of 2.5–5 IU/ml do not appear to be sufficient to achieve an optimal antiviral effect. This limit was also highlighted by Jalkanen et al., who suggested to use the parenteral route in order to achieve more efficacious concentrations (Jalkanen et al., 2020). Higher, more specifically pulmonary concentrations could be obtained with inhaled IFN, but available pharmaceutical forms and pharmacokinetic data on this method of administration remain limited in the Western world (Aricò et al., 2020).

### Impact of Type I Interferon Nebulization on COVID-19 Patients: Experience From China

Pulmonary administration of type I IFN may have the advantage of significantly reducing systemic adverse effects, while increasing its concentration in the infected epithelium (Mary et al., 2020). The Chinese Center for disease Control and Prevention (China CDC) early proposed the use of IFN-α-2b to treat COVID-19, as it has been historically used in China to treat viral pneumonia associated with SARS-Cov and middle east respiratory syndrome coronavirus (MERS). The first published data in favor of the use of inhaled IFN-α-2b date from February-March 2020 (Mary et al., 2020). First Liu et al. suggested that combination therapy involving IFN-α-2b inhalation combined with lopinavir/ritonavir and low-dose corticosteroids contributed to the observed 0% mortality in their COVID-19 patients, but without reporting any numerical data (Liu et al., 2020). More recently, the retrospective cohort from Chongqing public Health Medical Center presented the same 0% mortality in 217 patients (including 34 with severe forms), of which 99.5% were treated with therapy including IFN-α-2b (Ouyang et al., 2020). In the retrospective cohort study from Tongji University, there was a significant association between IFN-α-2b use and patient survival (OR2.32 IC95% [1,36; 3,97]) (Chen T. et al., 2020). Subsequently, reports have reinforced the potential of nebulized IFN to reduce mortality and viral carriage. Thus in Wuxi, China zero mortality was also observed in 55 COVID-19 patients, including 8 with a severe form of the disease, using a strategy including IFN-α-2b nebulization (Jiang X. et al., 2020), while in Wuhan, China a retrospective study performed in four different hospitals (including Tongji) found an independent association between IFN-α-2b inhalation and survival (3.45 [95% CI 1.96–0.51 5.88], *p* < 0.001) (Mei et al., 2020). In Anhui, China the early use of IFN-α-2b was independently associated with a reduction of viral carriage duration of SARS-CoV-2 (HR 1.649; 95% CI, 1.162–2.339) (Zuo et al., 2020). In Wuhan, China Zhou et al. showed in collaboration with E. Fish (Canada) that IFN-α-2b inhalation therapy combined with arbidol was associated with a significantly shorter duration of viral shedding compared to patients receiving arbidol alone (21 vs. 28 days; *p* = 0.002). Inhaled IFN-α-2b with or without arbidol also decreased serum IL-6 and CRP concentrations (Zhou et al., 2020). However, the initial characteristics of the patients (age, comorbidity, days from symptom onset to treatment) differed among the groups making any firm conclusion difficult (Zhou et al., 2020).

In other studies using inhaled IFN-α-2b, relatively short shedding times have been reported such as respectively in Shenzhen, China for moderate forms of COVID-19 (percentage of SARS-CoV-2 negative shift: 66% on day 6 and 95% on day 15) (Yuan J. et al., 2020) and in Shanghai, China for mild forms (90% on day 7) (Chen J. et al., 2020). Of note, the addition of other therapeutic agents to IFN-α-2b nebulization did not result in significant changes in viral carriage time, be it hCQ (Chen J. et al., 2020), ribavirin (Yuan J. et al., 2020) or arbidol (Xu et al., 2020). Finally, in a prospective trial carried out in Hubei, China the prophylactic administration of IFN by the nasal route combined with barrier measures was reported to lead to a zero incidence of COVID-19 among caregivers (Meng et al., 2020). The interest of IFN was reinforced by a study carried out in Changsha, China where the administration by inhalation of Novaferon (an non-natural IFN, modified to gain affinity) in an open randomized trial conducted in 89 hospitalized COVID-19 patients led to a faster reduction of viral carriage than lopinavir alone (with a median viral carriage time from nine down to 6 days), and no transition to a severe disease form in the Novaferon group, as compared to 14% in the lopinavir group alone (Zheng et al., 2020). More recently, Fu et al. reported that aerosol inhalation of type I IFN-κ plus TFF2 (a healing and anti-inflammatory polypeptide) in combination with standard care is safe and superior to standard care alone in shortening the time up to viral RNA negative conversion in patients with moderate COVID-19 (Fu et al., 2020).

Collectively, the encouraging results obtained with IFN-α-2b in COVID-19 patients in China, together with the complete absence of IFN-β release, the greater sensitivity of SARS-CoV-2 to IFN-β *in vitro* and the fact that auto-antibodies against IFN-β appear to be rare in severe COVID-19 patients gave a reason for hope regarding superior clinical efficacy of inhaled IFN-β.

### Current Data on the Safety of Inhaled Type I Interferon

Early studies on type I IFN nebulization showed that it was necessary to exceed 18 MIU (Million International Unit) of IFN-α-2b and 100 MIU of IFN-β to reach detectable concentrations of these compounds in blood (Halme et al., 1994). Similar data were obtained in non-human primates who exhibited acceptable local tolerance after direct instillation of 12 MIU of IFN-β-1a into the lungs and developed only mild subchronic alveolitis (Martin et al., 2002). This relatively good tolerance in preliminary studies was encouraging.

The only complete, currently available Treatment Emergent Adverse Events (TEAE) study with IFN-β inhalation comes from Synairgen, a drug discovery and development company founded by the University of Southampton, United Kingdom who developed SNG001, an aerosolizable form of IFN-β-1a. SNG001 has been used in phase 1 and 2 clinical trials with the aim to reduce rhinovirus-related symptoms in asthma patients. The drug allowed limited use of corticosteroids and presented few side effects (limited to 6.9% heart palpitations) (Djukanović et al., 2014). Moreover, no fever was observed although it was found in almost half of the patients when the product was administered subcutaneously (Limmroth et al., 2011). Psychiatric side effects were limited to rare sleep disturbances, and there was no significant hematological or hepatic toxicity, consistent with poor systemic passage of IFN aerosols (Djukanović et al., 2014). In view of this favorable risk/benefit balance, we decided to develop a nebulization protocol for IFN-β-1b administration. We present here the methods of IFN-β-1b administration, and the results obtained in four patients after administration on a compassionate basis.

## Characteristics of the Solution Devised to Nebulize Interferon-β-1b

### Choice of Solvents and Excipients

Many excipients contained in commercially available IFN formulations are at high risk of irritation. Well-known examples are acetic acid (Amdur, 1961) and benzyl alcohol (Wibbertmann et al., 2000). On the European market, only two type I IFNs have excipients which are fully compatible with the inhalation route: Betaferon^®^ (marketed by Bayer Healthcare) and its biosimilar Extavia^®^ (marketed by Novartis) which are both IFN-β-1b agents. These formulations are free from irritant compounds and contain albumin as well as mannitol, both of which are excipients recommended for dry inhalation (Bosquillon et al., 2001; Chew and Chan, 2002). Experiments presented in this work were undertaken with Extavia^®^. Extavia^®^ is sold with a vial of 1.2 ml of solvent containing 0.54% NaCl that is necessary to reconstitute the product. Considering China CDC’s recommendation to use 2 ml of water for injection (WFI), and the aqueous formulation of SNG001, we considered WFI to be suitable as a solvent, without organoleptic properties, for IFN-β-1b.

### *In Vitro* Assessment of Stability

**FIGURE 2:**
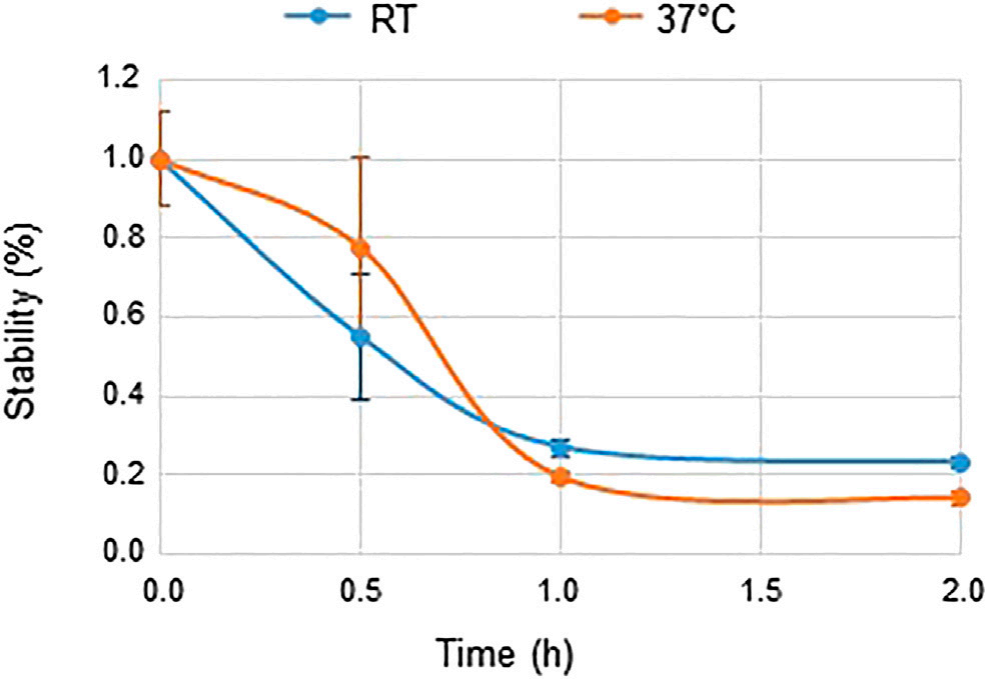
Stability of IFN-β-1b. Results represent the mean ± SD of three experiments performed in duplicate. Two way ANCOVA. Time effect, *p* < 0.0001. Temperature effect, *p* = 0.82. RT: Room Temperature 20°C.

The stability of IFN-β-1b was tested at 30 min, 1 h and 2 h after reconstitution in 2 ml of WFI. Experiments were performed either at room temperature (RT, 20°C) or at 37°C. The concentration of IFN-β-1b was determined by immunoassay (R&D systems: DY008) according to the manufacturer’s recommendations. The absorbance was read by spectrophotometry at 450 nm. When reconstituted at RT, a rapid decrease in IFN-β-1b mass was observed in the solution (Figure 2). Specifically, 45% of the product was lost 30 min after reconstitution at RT. Stabilization was observed 1 h after reconstitution with only 25% of IFN-β-1b remaining in the solution. Same data were obtained when the product was reconstituted at 37°C (Figure 2).

### Nebulization of Interferon-β-1b in a Cascade Impactor: Studies of Particles Size, Content and Distribution

**FIGURE 3:**
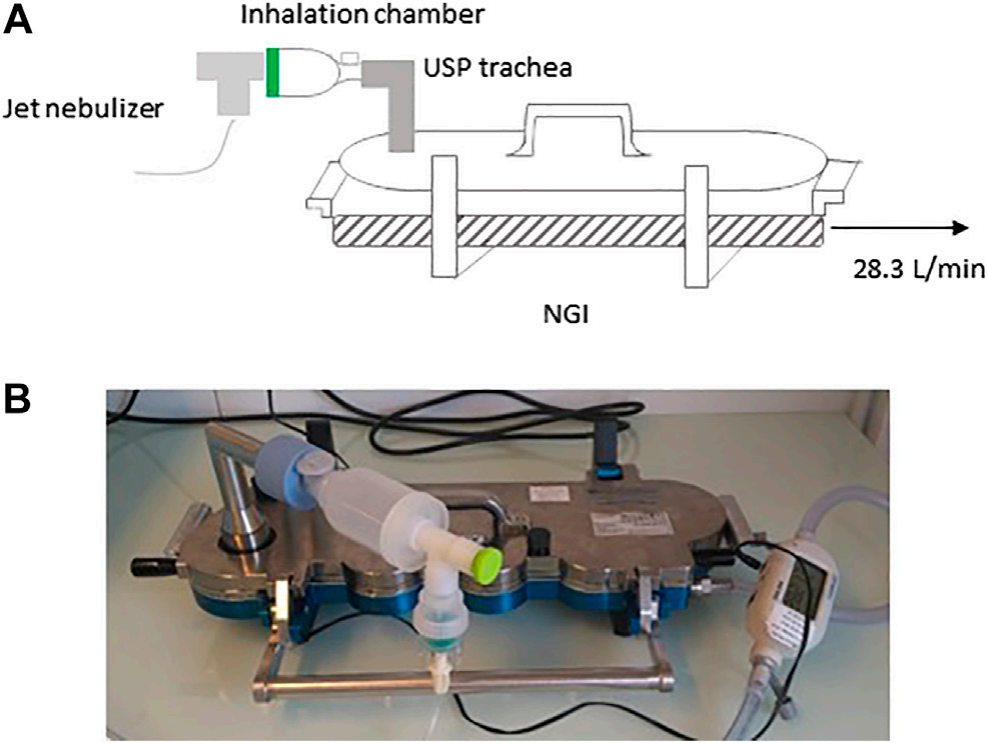
Schematic representation **(A)** and picture **(B)** of the NGI cascade impactor used to determine particle size of IFN-β-1b. NGI, Next Generation Impactor.

To determine whether the size of IFN-β-1b-containing aerodynamic particles is compatible with deposition in the bronchial tree, the solution of IFN-β-1b reconstituted in 2 ml WFI was nebulized in a new generation cascade impactor (NGI, Copley Scientific). Particle size, content and distribution were then studied. To do so, the impactor was connected to the USP (United States Pharmacopeia) trachea and the HCP5 pump (Copley Scientific) at a constant flow rate of 28.3 L/min according to USP <1602>. Leakage was checked using a 4,043 flowmeter (Copley Scientific) before each series of measurements. The measured flow rate did not exceed a 5% error from the desired flow rate. A 10-min nebulization was performed with jet nebulizer MICROMIST^®^ (Hudson RCI, ref. 41,745). A schematic representation of the assembly is depicted in Figure 3. Inside each impactor stage, trachea, and nebulizer chamber, the amount of IFN-β-1b (deposition or residual quantity) was determined by immunoassay (R&D systems: DY008) according to the manufacturer’s recommendations. The absorbance was read by spectrophotometry at 450 nm.

**FIGURE 4:**
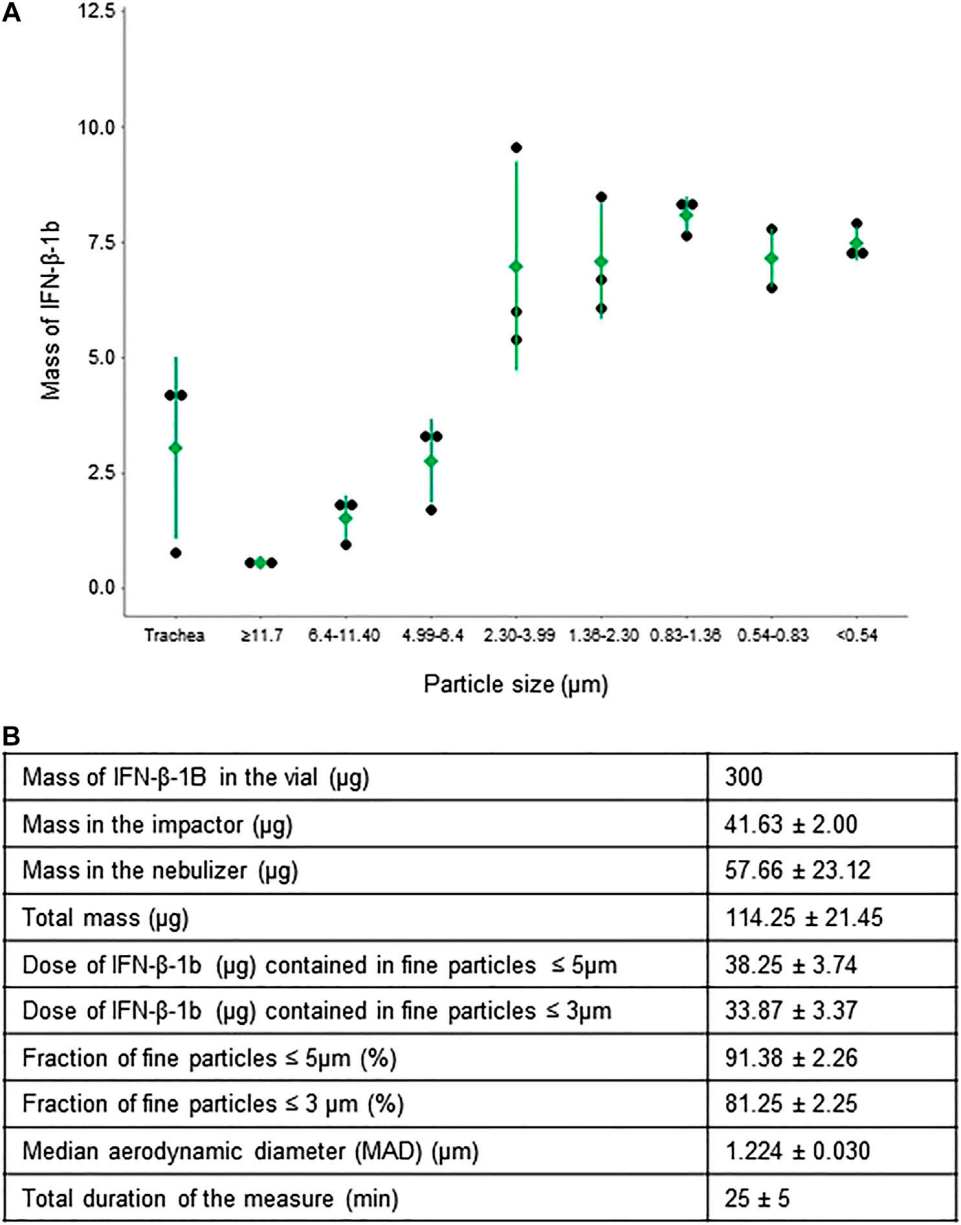
Distribution of IFN-β-1b in fine particles. **(A)** Mass of IFN-β-1b in particles of different aerodynamic sizes. Means ± standard deviation are shown in green. Each black dot represents one experiment (*n* = 3). **(B)** Mass of IFN-β-1b in fine particles and in residue. Data are expressed as mean ± standard deviation (*n* = 3). Total duration of the measurements: 25 ± 5 min.

The characteristics of the particles obtained by jet nebulizer are shown in Figure 4. Despite an initial quantity of 300 μg, only 114.25 ± 21.45 µg (mean ± SD) of IFN-β-1b was detected in the entire set-up after nebulization, consistent with the previously observed stability reached at 30 min. Although at the end of the 10 min nebulization no residual volume was visible in the nebulization chamber, there were still 57.66 ± 23.12 µg (mean ± SD) of IFN-β-1b remaining in the chamber, suggesting IFN-β-1b adhesion to chamber surfaces.

Inside the impactor we measured 41.63 ± 2.00 µg of IFN-β-1b, with a majority of fine particles ≤5 µm (91.38 ± 2.26%), and a mean particle size of 1.24 µm. Particle sizes between 4 and 0.54 µm (compatible with alveolar and bronchial deposition) accounted for the majority of the aerosolized IFN-β-1b, i.e., 32 µg (Figure 4). The jet nebulizer also allowed some deposition in the upper respiratory tract, of around 2.5 µg. In summary, our IFN nebulization technique allowed a topical delivery directly into the lungs of at least 10% of the total administered dose. In parallel to these *in vitro* studies, we have been able to treat four patients suffering from severe COVID-19 with IFN-β-1b inhalation, thereby providing an initial clinical feedback (see below).

## Cases-Report: Compassionate Application of Interferon-β-1b Inhalation to four Intensive Care Patients With Severe COVID-19

Given the therapeutic rationale for IFN-β-1b aerosol, and the first results in favor of an acceptable particle size for local deposition in the lungs, an intervention consisting in the inhalation of IFN-β-1b for the treatment of four patients with a severe form of COVID-19 was suggested by the clinical pharmacist to the intensive care unit team. Consent of the patients or their support person was obtained before proceeding to this off-label therapy and informed written consent of the patients was obtained to collect retrospectively individual data to follow CARE guidelines. Table 1 presents the demographic and clinical characteristics of the four patients on critical care admission.

**TABLE 1:** Demographic and clinical characteristics of patients on critical care admission.

	Patient 1	Patient 2	Patient 3	Patient 4
Age (years)	59	56	66	56
Sex	Male	Female	Male	Male
Body mass index	42.9	39.3	27.3	25.3
Medical history	T2DM (NID) dyslipidemia	Hypothyroidism dyslipidemia	Total thyroidectomy T2DM (NID) schizophrenia	Benign prostate hyperplasia
Symptoms on admission	Atrioventricular block fever	ARDS fever	ARDS collapsus oligoanuria tachycardia fever	Abdominal pain acute peritonitis fever

T2DM, type 2 diabetes mellitus; NID, non-insulin dependent; ARDS, acute respiratory distress syndrome.

**FIGURE 5:**
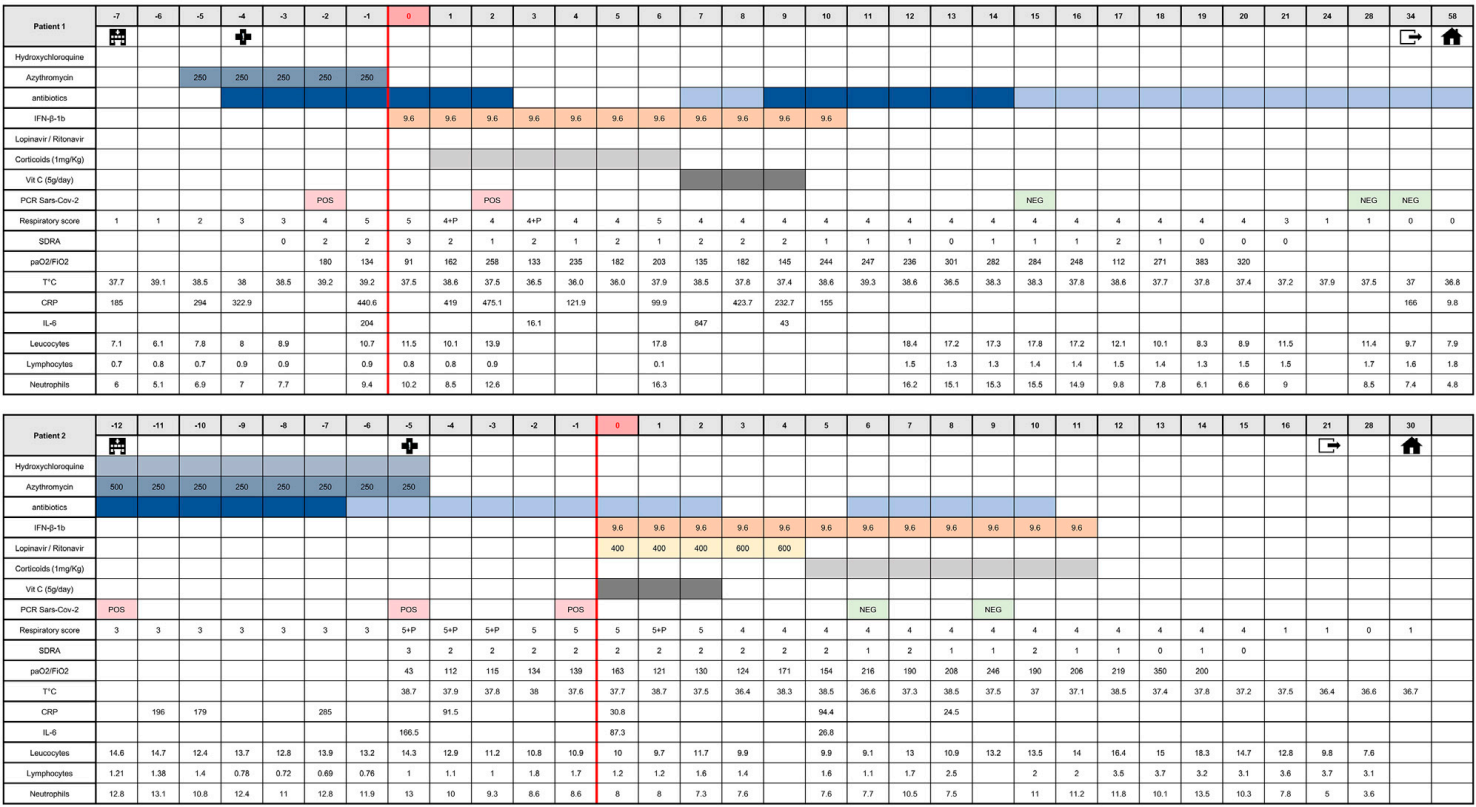
Medical history of patients one and two from admission time to hospital discharge. This table shows clinical course of major symptoms and signs, blood biochemistry and viral shedding on initial therapy (azithromycin and/or hydroxychloroquine) as well as changes of these parameters upon start of IFN-β-1b therapy until hospital discharge. Respiratory scores: 0, No oxygen; 1, oxygen through nasal cannula or mask; 2, mask with high O2 concentration; 3, optiflow and/or NIV (Non-Invasive Ventilation); 4, mechanical ventilation with PEP ≤ 6 cm H2O or FiO2 ≤ 0.6; and 5, mechanical ventilation with PEP > 6 cm H2O and FiO2 > 0.6. *p*, prone therapy (18 h). ARDS scores: 0, no ARDS; 1, light ARDS; 2, moderate ARDS; 3, severe ARDS (corresponding to Berlin ARDS definition). CRP, C-reactive protein; ARDS, acute respiratory distress syndrom; T°C, temperature; G, giga.

**FIGURE 6:**
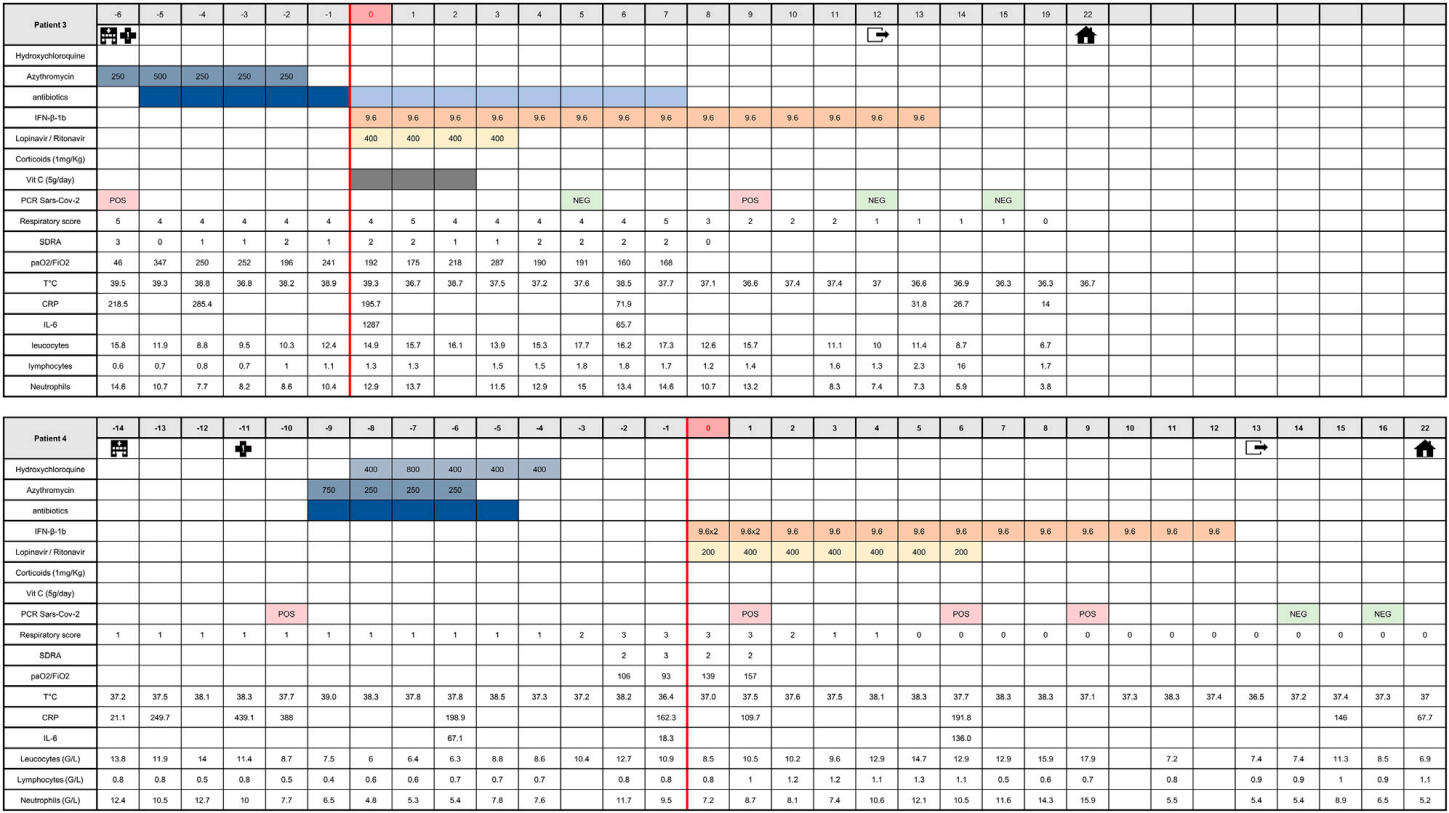
Medical history of patients three and four from admission time to hospital discharge. This table shows clinical course of major symptoms and signs, blood biochemistry and viral shedding on initial therapy (azithromycin and/or hydroxychloroquine) as well as changes of these parameters upon start of IFN-β-1b therapy until hospital discharge. Respiratory scores: 0, No oxygen; 1, oxygen through nasal cannula or mask; 2, mask with high O2 concentration; 3, optiflow and/or NIV (Non-Invasive Ventilation); 4, mechanical ventilation with PEP ≤ 6 cm H2O or FiO2 ≤ 0.6; and 5, mechanical ventilation with PEP > 6 cm H2O and FiO2 > 0.6. *p*, prone therapy (18 h). ARDS scores: 0, no ARDS; 1, light ARDS; 2, moderate ARDS; 3, severe ARDS (corresponding to Berlin ARDS definition). CRP, C-reactive protein; ARDS, acute respiratory distress syndrom; T°C, temperature; G, giga.

The medical history of patients 1 and 2 and patients 3 and 4 from time of hospital admission to time of hospital discharge is shown in Figures 5, 6, respectively. Before treating the patients with inhaled IFN-β-1b, particle size was checked *in situ*, directly in patient’s room, using an inverted microscope (Leica DMIL). Observed particles size was between 1.5 and 4.5 µm (Supplementary Figure S1).

### Demographic and Clinical Characteristics of Patients on Critical Care Admission

The four patients (three men and one woman), aged 56–66 years, were admitted to the intensive care unit between april 4 and april 12, 2020. The main reason for admission was COVID-19 pneumopathy for patients 2 and 3 (Table 1). All of them presented fever >38°C at admission (Table 1). Patient one was admitted for decompensation of an atrioventricular block, which required emergency implantation of a pacemaker. Patient four was admitted with Grade E acute pancreatitis of biliary origin with criteria of a Systemic Inflammatory Response Syndrome, and the notion of a 48-h exposure to a room neighbor who proved to be SARS-CoV-2 positive. No patient presented chronic kidney disease or hypertension, and none reported smoking. The diagnosis of COVID-19 was made rapidly after ICU admission, except for patient two whose diagnosis was made prior to transfer (Figures 5, 6). The diagnosis was confirmed by both RT-PCR on nasopharyngeal swab and chest CT. The RT-PCR method was performed based on recommendations by the National Reference Center for Respiratory Viruses, Institut Pasteur, Paris, using nCoV_IP2 and CoV_IP4 sequences (Institut Pasteur, 2020). The CT scans showed characteristic images of SARS-CoV-2 infection including bilateral frosted glass opacities of the lung parenchyma for all and bilateral pleural effusion for patient 4. On entry into critical care, all patients also had serum CRP levels >100 mg/L, and abnormal white blood cell counts with lymphocytopenia.

### Initial Strategy of Care

The decision to treat these patients with IFN-β-1b was taken because they did not meet the inclusion criteria of other ongoing clinical trials such as Discovery (cardiac contraindication, or RT-PCR > 72 h). The initial strategy of care for these patients respected the recommendations made during this period in France. For severe forms of COVID-19, the HCSP (Haut Conseil de Santé Publique) suggested considering (case by case and according to a collegiate body’s opinion) either the use of lopinavir/ritonavir or hCQ with close cardiological monitoring (HCSP, 2020). Only patient 2 started treatment targeting Sars-CoV-2 before she went into intensive care. At the time of COVID-19 diagnosis, lopinavir/ritonavir could not be prescribed to these patients because the product was out of stock. Instead all patients received antibiotic coverage with cefotaxime and azithromycin for severe community-acquired pneumonia. This treatment was combined with hCQ for patients 2 and 4, but not for patients 1 and 3 for whom the use of hCQ was contraindicated. Oxygen therapy was regularly adapted to the need of each patient, combined with 18 h prone therapy for patients 1 and 2 (Figures 5, 6).

Despite this management, the respiratory status of all patients deteriorated, with the appearance of severe ARDS with paO2/FiO2 <100 mmHg according to Berlin criteria. Patient 4 received high-flow oxygen therapy (Optiflow^™^) > 50 ml/min, considered as an application of PEEP >5 mmHg (Chertoff, 2017).

Due to marked respiratory status deterioration of these four patients, the medical team proposed to start a treatment with inhaled IFN-β-1b.

### Inhalation of Interferon-β-1b and Associated Therapies

IFN-β-1b (reconstituted with 2 ml WFI) was administered with MICROMIST^®^ jet nebulizer at a dosage of 9.6 MIU (entire vial containing 8 MIU with 20% overage to facilitate reconstitution) per day, with a loading dose at 9.6 MIU bid for 48 h given to patient 4. Concomitantly, lopinavir/ritanovir (with dosage adjustment to serum concentrations) was administered to patients 2, 3 and 4 (Figures 5, 6). The main data in favor of the recommendation to use lopinavir/ritonavir in hospitalized patients came from a report by Cao et al. (Cao et al., 2020). In front of the general deterioration of the patients' health, and given the availability of lopinavir/ritonavir at the time when patients 2, 3, and 4 entered the compassionate protocol, the team decided to follow the Shenzen recommendations to associate corticosteroids (for patients with a high inflammatory status), lopinavir/ritonavir and inhalation of IFN in order to improve the chance of survival (Liu et al., 2020). Patient 1 did not receive lopinavir/ritonavir because the product remained out of stock when he entered the ICU one week before patients 2, 3, and 4. Vitamin C (5 g/day for 3 days) as anti-oxidant therapy was given to patients 1, 2, and 3. Methylprednisolone (1 mg/kg) as anti-inflammatory therapy was administered to patients 1 and 2 (Figures 5, 6). All patients also received anticoagulation therapy by sodic heparin (curative dose) during resuscitation. Patient 1 subsequently received argatroban due to heparin-induced thrombocytopenia. IFN-β-1b aerosol treatment was administered for 10–14 days.

### Impact of Interferon-β-1b Inhalation on Major Clinical Parameters, Blood Biochemistry and Viral Shedding

The comparison of clinical and biochemical parameters between start of IFN-β-1b treatment and after 15 days of management is presented in Table 2. All patients showed an improvement in respiratory status, with only one patient still meeting mild ARDS criteria. Two patients were extubated, and in particular patient 4, after his loading doses, showed a resumption of respiratory secretions which allowed mobilization by physiotherapy, followed by early respiratory weaning, when the patient’s intubation was about to be performed. Such rapid resumption of pulmonary mucus secretion following inhalation of IFN-β-1b was also observed in patient 1. It could not be evaluated in the two other patients who had a bacterial infection with purulent secretions.

**TABLE 2:** Impact of IFN-β-1b inhalation on major symptoms, duration of viral shedding and outcomes in four patients hospitalized with COVID-19. If values were not available at d15 and d-1, the closest values available before treatment and toward the end of treatment.

IFN-β-1b therapy	Patient 1		Patient 2		Patient 3			Patient 4	
	d-1	d15	d-1	d15	d-1		d15	d-1	d15
Respiratory score	5	4	5	4	4		1	3	0
ARDS	2	1	2	0	1		0	3	0
T°C	39.2	38.3	37.6	37.2	38.9		36.3	36.4	37.4 (d12)
CRP	440.6	145 (d10)	91.5 (d-4)	24.5 (d8)	285.4 (d-4)		26.7 (d14)	162.3	146
IL-6	204	43 (d9)	166.5 (d-5)	26.8 (d5)	1,287 (d0)		65.7 (d7)	18.3	136.0 (d6)
Leukocyte count (G/L)	10.7	17.8	10.9	14.7	12.4		8.7 (d14)	10.9	11.3
Lymphocyte count (G/L)	0.9	1.4	1.7	3.1	1.1		1.6 (d14)	0.8	1
Neutrophill count (G/L)	9.4	15.5	8.6	10.3	10.4		5.9 (d14)	9.5	8.9
First negative PCR	Day 15		Day 6			Day 12		Day 14	
Discharge from resuscitation	Day 34		Day 21			Day 12		Day 13	
Discharge from home	Day 58		Day 30			Day 22		Day 22	

For all patients, the respiratory score improvement was concomitant with normalization of body temperature (Table 2). The treatment with inhaled IFN was accompanied by a decrease in serum CRP in all patients. Serum IL-6 also decreased except for patient 4, and was fluctuating in patient 1, according to the use of continuous venous dialysis. At the same time, the white blood cell count normalized, with an increase in lymphocyte count except for patient 4 (Table 2). Patients received regular nasopharyngeal swabs, and negative portage was found in all after two successive negative PCRs, after 6–15 days of management.

In terms of other hospitalization complications, patient 1 had acute kidney failure requiring continuous veno-venous dialysis, and developed heparin-induced thrombocytopenia. Two patients had secondary infections requiring antibiotic therapy on a probabilistic or documented basis (*Citrobacter Koseri* pneumonia and methicillin resistant *Staphycoccus epidermidis* bacteremia in patient 1, and ventilation-acquired *Serratia marcescens* pneumonia in patient 3). Patient 2 had his broad-spectrum antibiotic coverage discontinued upon return of negative bacteriology results.

None of the patients deteriorated further, and after 13–34 days they were all discharged from intensive care. Oxygen withdrawal was achieved during hospitalization in three among the four patients. At 3 months’ follow-up, all patients were alive.

## Discussion

We were able to treat with inhaled IFN-β-1b, on a compassionate basis, four patients with severe COVID-19 admitted in the intensive care unit. Neither antibiotic nor hCQ administration had provided a satisfactory therapeutic response in these patients with severe co-morbidities. They showed an improvement in clinical and biochemical parameters within an acceptable time frame, in the absence of treatment-related adverse events. All of them were discharged from hospital and subsequently resumed normal life after a period of rehabilitation. Based on these observations, it seems reasonable to assume that inhaled IFN-β has exerted an antiviral action by restoring deficient pulmonary innate immunity defenses. This hypothesis is strongly supported by very recent immunological data supporting a role of insufficient response of type I IFNs (in particular with absence of IFN-β secretion) in the severity of COVID-19 (Arunachalam et al., 2020; Blanco-Melo et al., 2020; Hadjadj et al., 2020). The rationale of this treatment is further supported by the efficacy of IFN against SARS-CoV-2, both *in vitro* and *in vivo,* based on evaluation in retrospective studies and three randomized trials (Davoudi-Monfared et al., 2020; Hung et al., 2020; Rahmani et al., 2020).

Although the use of the pulmonary route represents an asset in terms of therapeutic precision, lack of knowledge of IFN nebulization remains a major limitation (Aricò et al., 2020). In order to apply this therapeutic modality to COVID-19, we explored the possibility of nebulizing IFN-β-1b.

Our approach is currently unique. In China, several randomized clinical trials have been built around different forms of IFN-α-2b (Chen J. et al., 2020; Jiang X. et al., 2020; Liu et al., 2020; Mei et al., 2020; Zhou et al., 2020; Zuo et al., 2020). This approach is currently explored by Fish et al. in Canada with the development of AP003 (BetterLifePharma Inc) (Choudhury, 2020). The more direct approach of pulmonary IFN-β nebulization is presently explored by Wilkinson et al. in collaboration with Synairgen, in a clinical trial with SNG001, a nebulizable form of IFN-β-1a (NCT04385095). However, these approaches are moving toward patent-protected products, with limited supply. A main advantage of IFN-β-1b is its immediate supply as a biosimilar, with a formulation suitable for nebulization without the need of major modifications. IFN-β-1b differs from IFN-β-1a in the way it is produced. On the one hand, IFN-β-1b is synthesized by bacteria without glycosylation and with modifications of 2 amino-acids to maintain stability. IFN-β-1a, on the other hand, is produced by mammalian cells and has a structure identical to natural IFN-β. Their difference results in a decrease of pharmacodynamic activity of about 25% for IFN-β-1b, as compared to IFN-β-1a. The decreased activity is compensated by a higher dosage of IFN-β-1b per vial (Stürzebecher et al., 1999).

Using a cascade impactor, we found that our process allowed nebulization of approximately 1 MIU (32 µg) of IFN-β-1b along the bronchial tree. The distribution of particle sizes evenly distributed between 0.54 and 5.0 µm makes it possible to cover both alveoli and bronchioles (Cheng, 2014). The volume of pulmonary surfactant being limited from 1 to 5 ml, the administration resulted in a local epithelial concentration ranging from 10^6^ IU/ml to 2.10^5^ IU/ml. This amount allowed the achievement of a local concentration higher than that for which IFN has been shown to be most effective against SARS-CoV-2 (>50 IU/ml) (Clementi et al., 2020) for at least 12 local half-life. These concentrations are well above the 2.5 IU/ml cmax achievable by the subcutaneous (SC) pathway (Greig et al., 1988; Salmon et al., 1996; Munafo et al., 1998). The SC route of administration was chosen in the Discovery and Solidarity trials before knowing the EC50, and the ideal targets for SARS-CoV-2. The SC route does not appear to be optimal because it exposes to more adverse effects (Djukanović et al., 2014), and does not achieve full virucidal concentrations observed in a curative model (Clementi et al., 2020). The SC route also exposes to an increased risk of neutralizing autoantibodies against IFN-β, which could compromise the efficacy of IFN-β-based therapeutics as previously described in patients with multiple sclerosis (Sominanda et al., 2010). The pulmonary route may help to overcome this issue. The action of the aerosol is mainly localized at the level of the epithelium, with uncertainties regarding the amount that diffuses into lung parenchyma. Nevertheless, in the context of SARS-CoV-2, this local effect is particularly adapted to the marked tropism of the virus for epithelial cells, compared to other respiratory viruses (Hui et al., 2020). The deposited amount of IFN-β-1b (although representing only 10% of the initial content) therefore appears to be sufficient for a topical pulmonary effect. This is consistent with the rapid pharmacodynamic effect observed in two of the four patients, with the resumption of bronchial mucus secretion.

Galenic optimization of dosage and composition would be welcome to use lower amounts of IFN-β-1b (produced by genetic engineering). For example with IFN-γ, the choice of nebulizer and the use of feedback systems can heavily influence the amount deposited in the lung, between 7 and 65% (Condos et al., 2004; Diaz et al., 2012; Sweeney et al., 2019). Beyond the choice of the nebulizer, we are also faced with the problem of stability of the reconstituted product. By analogy, IFN-α in solution is known to aggregate and adhere to surfaces, limiting monomeric forms to 25% after 25 min (Ip et al., 1995). Ionic strength did not influence this aggregation, (Ip et al., 1995). The aggregation constitutes a pre-existing constraint for IFN-β-1b, with an aggregation rate of about 15% upon reconstitution (Barnard et al., 2013). Galenic optimization for protein nebulization is complex, and could be achieved by different excipients such as PEG 8000, n-Dodecyl-β-D-maltoside, l-arginine and trehalose (Mahjoubi et al., 2015; Sécher et al., 2019). The use of pegylated IFNs could also be considered to both increase stability and extend action at the local level (Mcleod et al., 2015).

The issue of galenic optimization is closely linked to the optimization of administered doses to keep antiviral lung IFN concentration over time. In order to be more effective at treatment start, we propose inhalation bid for the first 48 h. The patient who received such high dosing was the one who had the best clinical outcome. In hepatitis C, the anti-viral action of IFN-β has been shown to be dose-dependent and more pronounced over 48 h, which reinforces the concept of the need for more aggressive dosing at treatment initiation (Hosseini-Moghaddam et al., 2009). However, pharmacokinetic studies are required to evaluate local concentrations, as well as the elimination time of the product to better determine the optimal dosing regimen.

Our four patients had all moderate to severe ARDS at the time of treatment initiation and improved respiratory function upon inhalation of IFN-β-1b. At 3 months of follow-up they were all alive. The probability of such a good outcome was estimated at 2.5–32%, considering an intensive care mortality of 25–60%. One should however also take into account the overall optimization of patient management, in particular anticoagulation (Atallah et al., 2020), frequent use of glucorticoids (RECOVERY Collaborative Group et al., 2020a) and vitamin C (Carr, 2020; Colunga Biancatelli et al., 2020), in these highly inflamed individuals, as well as an organization allowing prone therapy (Coppo et al., 2020). Based on recently published results from the Recovery study, it is unlikely that lopinavir/ritonavir contributed to patient improvement (RECOVERY Collaborative Group, 2020). However, the existence of a synergistic effect between IFN-β-1b and loponavir/ritonavir cannot be completely ruled out. In the same manner, given the recent data showing that the use of hCQ and/or azithromycin does not improve clinical outcomes in hospitalized patients (RECOVERY Collaborative Group et al., 2020b; Fiolet et al., 2020; Furtado et al., 2020), it is unlikely that the initial therapy given to these patients contributed to the observed clinical improvement. Although it is not possible to draw a firm conclusion on treatment efficacy based on our preliminary observations the safety and tolerance of IFN-β-1b inhalation are reassuring. Particular attention should be paid to the risk of secondary bacterial infections, especially in case of prolonged treatments (Boxx and Cheng, 2016). In addition, IFN therapy may have diminished efficacy or even become deleterious if administered too late against SARS-CoV-2 (Wang et al., 2020). Monitoring of inflammation and viral elimination therefore appears to be important parameters for individual adaptation of treatment.

In order to know the risk-benefit ratio of IFN-β-1b nebulization in the treatment of COVID-19 it is now important to evaluate this ratio in randomized controlled trials. Since IFN-β can act in both the viral and the inflammatory phases of COVID-19 further studies should evaluate the potential of inhaled IFN-β in all phases of the disease. The effect of SNG001 (inhaled IFN-β-1a) is currently evaluated in patients with no need of resuscitation. Our protocol was submitted to and approved by the regulatory authorities in France. It is registered under No. NCT04469491. We plan to include more patients with moderate and severe COVID-19 who are hospitalized and need oxygen therapy, whether in intensive care units or not.

## Data Availability Statement

The raw data supporting the conclusions of this article will be made available by the authors, without undue reservation.

## Ethics Statement

Ethical approval was not provided for this study on human participants because the results present medical record data, retrieved retrospectively. To follow CARE guidelines, Patients gave their written and informed consent to the use of their medical data to investigate the hypothesis described in the article. The patients/participants provided their written informed consent to participate in this study.

## Author Contributions

AM, LH, and MB developed the initial hypotheses and wrote the manuscript. AM, PYM, LB, and HD validated the proposed therapeutic approach and critically reviewed the manuscript. AM and AC were in charge of clinical data collection and microscopically checked particle size *in situ*. TP and ME performed *in vitro* experiments on the feasibility of IFN-β-1b nebulization. AM and LH designed the figures and tables.

## Funding

This project has not received any funding. The work was carried out in collaboration with Protecsom’s research and development department.

## Conflict of Interest

AM declares travel and accommodation support from Pfizer. These funders had no role in study design, data collection and analysis, decision to publish, or preparation of the manuscript. The remaining authors declare that the research was conducted in the absence of any commercial or financial relationships that could be construed as a potential conflict of interest.
