# Supplementary material for: Rationale for COVID-19 Treatment by Nebulized Interferon-β-1b–Literature Review and Personal Preliminary Experience
Source: Front Pharmacol. 2020 Nov 30;11:592543. doi: 10.3389/fphar.2020.592543 (PMC7734101; doi:10.3389/fphar.2020.592543)
Supplement: Supplementary file 1 [file datasheet1.pdf]

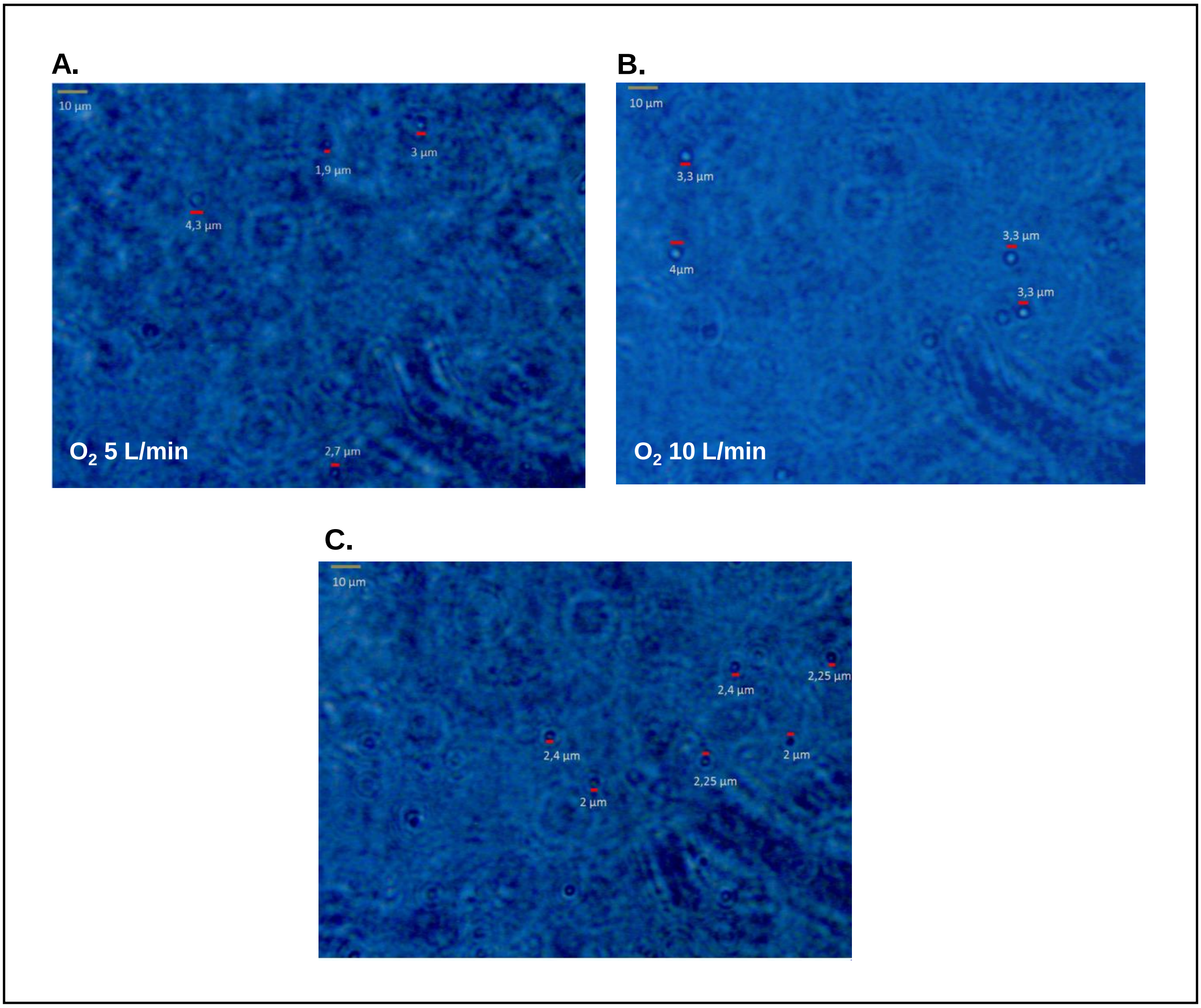

Supplemantal Figure 1 : Microscopic observation of fine particles after IFN-β-1b nebulization. IFN-β-1b (Extavia® Novartis) was reconstituted in 2 mL WFI and extemporaneously aerosolized with jet nebulizer MICROMIST® Nebulizer with in-Line Neb Tee® with valve from HUDSON RCI® (reference: 41745) for 1 min. **A.** and **B.** Particles of IFN-β-1b aerosol generated without respirator with a fixed flow of oxygen in the pneumatic nebulizer chamber. **C.** Particles of IFN-β-1b aerosol generated with a respirator using a pneumatic nebulizer (Drägher Evita XL ventilator). Particle diameter is shown in red. Magnification x40. n=3
